# Discovery of a Sweet Spot on the Foot with a Smart Wearable Soccer Boot Sensor That Maximizes the Chances of Scoring a Curved Kick in Soccer

**DOI:** 10.3389/fphys.2018.00063

**Published:** 2018-02-13

**Authors:** Franz Konstantin Fuss, Peter Düking, Yehuda Weizman

**Affiliations:** ^1^Smart Equipment Engineering and Wearable Technology Research Program, Centre for Design Innovation, Swinburne University of Technology, Melbourne, VIC, Australia; ^2^Integrative and Experimental Training Science, Institute for Sport Sciences, Julius-Maximilians University Würzburg, Würzburg, Germany

**Keywords:** smart soccer boot, pressure sensor, sweet spot, dead spot, probability of scoring a goal, center of pressure, impact force, wearable technology

## Abstract

This paper provides the evidence of a sweet spot on the boot/foot as well as the method for detecting it with a wearable pressure sensitive device. This study confirmed the hypothesized existence of sweet and dead spots on a soccer boot or foot when kicking a ball. For a stationary curved kick, kicking the ball at the sweet spot maximized the probability of scoring a goal (58–86%), whereas having the impact point at the dead zone minimized the probability (11–22%). The sweet spot was found based on hypothesized favorable parameter ranges (center of pressure in x/y-directions and/or peak impact force) and the dead zone based on hypothesized unfavorable parameter ranges. The sweet spot was rather concentrated, independent of which parameter combination was used (two- or three-parameter combination), whereas the dead zone, located 21 mm from the sweet spot, was more widespread.

## Introduction

It was recently proposed that wearable sensor technology (“wearables”) aid optimizing athletes performance by providing feedback about monitored context-specific parameters (Düking et al., 2017). This approach was successfully implemented in different settings (Crowell and Davis, 2011; Windt et al., 2017). Yet, a field which received little attention is the kicking action and more precisely the foot-to-ball impact phase in soccer. This is surprising, since soccer is the most popular sport in the world, and improving kicking actions is often part of soccer training (Kellis and Katis, 2007). The lack of research on wearables analyzing the foot-to-ball impact phase surely is limited by the lack of available sensor technologies to access relevant parameters.

In soccer, the direct free kick is one possibility of scoring a goal, and up to 6.31% of all goals are scored in elite (female) soccer (Alcock, 2010). Another, more challenging technique is the curved kick, where a stationary ball follows a curved trajectory around a human wall formed by defensive players in order to hit the goal. However, before this technique can be improved optimally in individual soccer players, characteristics of an ideal curved direct free kick must be analyzed and established. From a biomechanical point of view, soccer kicks can be analyzed from several kinematic and kinetic aspects, i.e., the approach, the supporting leg, the kicking leg, joint velocities, and the foot-to-ball impact (Kellis and Katis, 2007; Lees et al., 2010). However, it can be argued that the foot-to-ball impact phase is the paramount aspect of the kick since it is the only time players can influence the speed, spin and direction of the ball. In general, very little research has been conducted on curved direct free kicks and there is no single study available that addresses the differences of successful and non-successful curved direct free kicks in the foot-to-ball impact phase. This issue partly arises from methodological limitations related to evaluating the foot-to-ball impact phase.

The kicking action and particularly the foot-to-ball contact is usually investigated kinematically, by using high-speed cameras or motion analysis systems with body segment markers (Barfield et al., 2002; Dichiera et al., 2006; Nunome et al., 2006; Ishii and Maruyama, 2007; Shinkai et al., 2008; Scurr and Hall, 2009; Ball, 2011). The data sampling frequency or frame rate ranges from 50 Hz (Dichiera et al., 2006) to 5 kHz (Shinkai et al., 2008). Force plates are only useful to capture the action of the support leg (Ball, 2011). EMG (electromyography) was employed for analysing the muscle activity during kicking (Bauer, 1983; Dorge et al., 1999; Orchard et al., 2001). The kick impact force was estimated or derived in two different ways. Ishii and Maruyama (2007) assessed the deformation of the ball with high-speed cameras (2.5 kHz), as the force is a power function of the deformation based on Hertzian contact mechanics. The force calculated was ~1,200 N. Shinkai et al. (2008) also used high-speed cameras (5,000 Hz) for estimating the velocity of the center of mass of the ball, the slope of which at time of peak deformation (±1 ms) corresponds to the peak acceleration of the ball. The product of the latter and the mass of the ball yields the peak impact force. The average peak force reported by Shinkai et al. (2008) amounted to 2,847 ± 538 N. In summary, the problem of kinematics is that impact force can only be estimated, if calculated from other parameters obtained from kinematic analysis, rather than measured directly. The center of pressure (COP) of the foot-to-ball impact phase, however, cannot be determined from close-up ultra-high-speed camera data accurately.

To the best of our knowledge, the only research using wearable sensor technology specifically aiming to analyse the foot-to-ball impact phase was performed by Hennig et al. (2009) who equipped two shoes (best and the worst shoes in terms of instep kicking accuracy out of five commercially available soccer shoes) with a Pedar (Novel GmbH, Munich, Germany) pressure distribution measuring insole located outside of the shoe upper (Hennig et al., 2009). The pressure was measured on every other sensor at a frequency of 571 Hz. From the pressure data, the summation center of pressure (COP) was calculated (Hennig, 2011), which was located more medially and more proximally in the shoe that delivered more accurate kicks. While providing meaningful results, from our perspective, transferability of these to practice is restricted by the high costs of the used wearable sensor technology whereby this technology cannot be made available for amateur athletes. However, these are likely the ones benefiting the most from biofeedback by wearables (Düking et al., 2017).

A pressure-sensitive wearable technology was recently developed with the purpose of analyzing players' kicking technique at the foot to-ball impact phase was developed (Weizman and Fuss, 2015a,b). This technology has several advantages over commercially available pressure sensor array systems: it is cheap (cheaper than the Pedar insole by a factor of ~100); highly accurate in terms of impact COP measurement (far more accurate than a Kistler force plate); samples the data at 2–2.5 kHz per sensor. The pressure-sensitive wearable technology can be incorporated into athletes' footwear (which we will call from now on the “Smart Soccer Boot”) to precisely measure the position of the COP and the magnitude of the impact force at each instance in time at the contact area between a player's kicking foot and the ball. The Smart Soccer Boot was originally developed for training purposes, specifically to monitor the training load of kicking.

The aim of this study is to use the Smart Soccer Boot for exploring the accuracy of curved kicks, evaluating the probability of scoring a goal, linking the chances of success to dynamic parameters obtained from the smart boot (such as impact force and location of the center of pressure), and analyzing whether there is a spot on the shoe (sweet spot) that maximizes the chances of success when kicking the ball. This leads to four hypotheses:

*Hypothesis 1*: there is *no significant difference between the measured dynamic parameters* (COPx, COPy, impact force) of all hits (scoring a goal) and those of all misses. The reason being that there is no single “sweet spot” on the shoe or foot that guarantees a success rate of 100% for scoring a goal. There might be ball-shoe or -foot contact spots or zones that offer more or fewer chances of scoring a goal with areas of average chances in between these specific spots. As the probabilities are distributed across these spots and the areas in between and as the chances at the specific spots are not exactly 100 or 0% either, the dynamic parameters associated with hits and misses might considerably overlap and therefore not exhibit a significant difference.

*Hypothesis 2*: There is a *favorable parameter range* that maximizes the probability of success as well as an *unfavorable parameter range* that minimizes this probability. A method for identifying such parameter ranges has to be developed, which this research is based on. Furthermore, parameters of all hits and all misses cannot be directly compared, but rather extreme cases such as successful kicks and parameters within the favorable range vs. unsuccessful kicks and parameters within the unfavorable range. This approach separates the data and is expected to result in significant differences between COP locations that provide more or fewer chances of scoring a goal. The COP locations, however, are seen as a continuum across increasing/decreasing probabilities of success, and their extremes locations are spots with maximum chances and minimum chances of scoring a goal.

*Hypothesis 3*: If there is a favorable/unfavorable parameter range, then the extreme COP location related to the favorable range constitutes a well-defined *sweet spot*. If there really is a spot that maximizes chances then this will be a “*sweet spot*,” the definition of which is the location of COP that maximizes chances of scoring a goal.

*Hypothesis 4*: If there is a sweet spot on the boot/foot, then there is also a *dead spot or zone*. The dead spot is a spot located differently from a sweet spot, whereas a dead zone is e.g., a ring around the dead spot if there are feasible contact points around the sweet spot. Otherwise, a sector of a ring could be found that minimizes the chances of scoring a goal if the ball-boot/foot contact is located within this dead zone.

The use of an accurate measurement device is indispensable for this task, which, naturally, must be in the form of a wearable device located at the medial and dorsal part of the foot. Although the Pedar insole (Novel GmbH, Munich, Germany) is wearable inside a shoe, wrapping it around a soccer boot (as done by Hennig et al., 2009; Hennig, 2011, for finding the average COP of two shoes with different kick accuracies) is difficult as it was designed to be worn inside a shoe for plantar pressure measurement. As such, a smart wearable device specifically designed for measuring the ball-to-boot or -foot impact force and COP with high accuracy (Weizman and Fuss, 2015a,b; Weizman, 2016) was used in this study.

The term “sweet spot” used in this paper is adapted from sports implements. In racquets, bats and clubs, hitting a ball with the sweet spot either maximizes the performance (increase in ball speed; e.g., power spot of tennis racquets), or minimizes the risk of overstrain injuries (node point that minimizes racquet vibrations, and center of percussion that minimizes the shock force at the hand; Fuss, 2011; Fuss et al., 2014). These features are not applicable to the “sweet spot” on a shoe, boot or foot; nevertheless, kicking a ball at the sweet spot hypothetically maximizes the player's performance by increasing the chances of scoring a goal.

## Methodology

### Smart soccer boot

**Figure 1:**
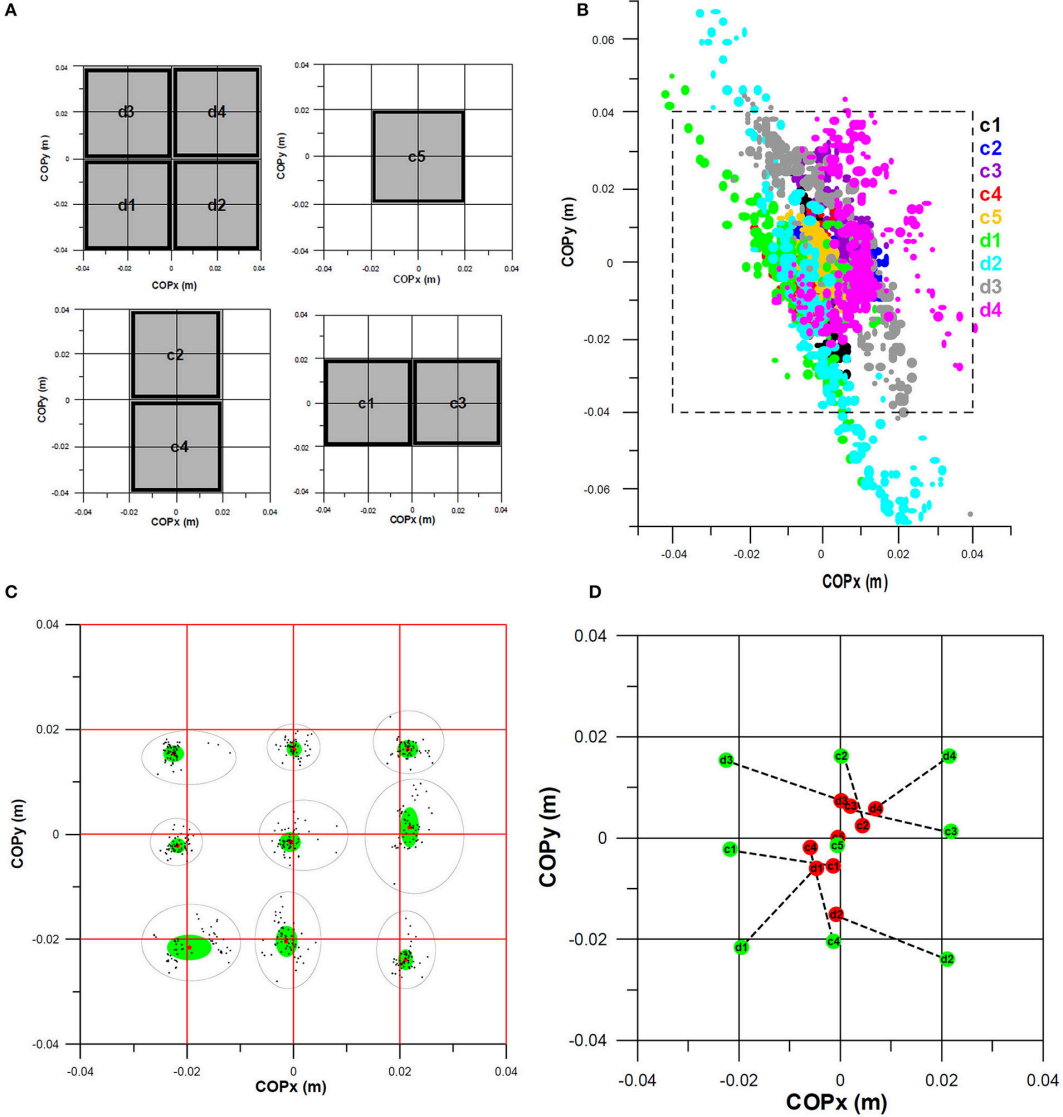
Pressure sensor matrix and its validation against a Kistler force plate; COPx, COPy: position of the center of pressure in x- and y-direction of the coordinate system of the sensor matrix; **(A)** 4 × 4 sensor matrix and the positions (d1–d4, c1–c5) of the spacers for impact loading of nine 2 × 2 quarters for validating the position of the center of pressure (COP); **(B)** COPs obtained from the force plate with respect to the sensor matrix (dashed black square; note that COPs cannot be outside the sensor matrix; yet, the force plate returned impossible COP positions); **(C)** COPs obtained from the pressure sensor matrix (black dots: COP position; red dots: average position; green ellipse: area of one standard deviation of COPx and COPy with respect to the average, black elliptic contour: cluster of all COP positions per quarter; note that the average COPs are not exactly at the center of each quarter as it was impossible to impart the impact force exactly at the center of each spacer); **(D)** Comparison of average COPs obtained from the force plate (red) and the pressure sensor matrix (green). ©Yehuda Weizman, reproduced from Weizman (2016) with kind permission.

The sensor array system for the Smart Soccer Boot (Weizman and Fuss, 2015a) consists of 16 sensor cells (Figure 1), a programmable microcontroller and a compact electronics circuit board. All sensor cells are arranged in a 4 × 4 matrix, whereby each cell is 20 × 20 mm separated by a 1 mm gap. The piezoresistive material used for the sensors consisted of an off-the-shelf piezoresistive vinyl, and exhibited a linear calibration curve when the pressure was plotted against conductance data derived from force impact tests (Weizman, 2016). Each sensor was calibrated individually and validated against a Kistler force plate (type 9260AA6, Kistler, Winterthur, Switzerland) with impact forces ranging from 368 to 2,146 N (Weizman and Fuss, 2015b). The *R*^2^ values when correlating measured sensor impact forces against measured impact force on the Kistler force plate ranged from 0.9333 to 0.9882 (0.9647 ± 0.0189; Weizman, 2016). The validation of the COP obtained from the force sensor against the one returned from the Kistler force plate failed, as the Kistler force plate was not able to measure the COP of impact forces accurately (Figure 1). In most cases, the COP obtained from the Kistler force plate was even outside the impacted sensors (impact on 4 adjacent sensor cells only, 2 × 2 matrix), even if the impact was confined to 4 sensors with a 10 mm thick wooden spacer, thereby preventing loading of adjacent areas (Weizman, 2016). The COP returned from the sensor was always very close to the center of the 4 sensor cells [“very close” because the impact force was applied manually and could not be centered precisely; (Weizman, 2016); Figure 1]. High precision in determining the center of pressure is paramount for the present study and its results.

### Participants

Ten right-footed and experienced male soccer players (*n* = 10; age = 26 ± 1.71 years; body height: 177.1 ± 5.43 cm; body mass: 75.2 ± 8.36 kg; shoe size [EU]: 43 ± 1.4) volunteered to participate in the study after having been extensively informed about all testing procedures. The recruited players were trained midfielders or strikers with at least 6 years of soccer training at a non-professional level.

The study was granted Ethics approval by the RMIT University Human Ethics Committee (approval no. ASEHAPP 28-14). All testings were carried out in accordance with the Declaration of Helsinki. No player suffered from injury, illness, and/or disease and all players were instructed to have eaten a light meal 1 h prior to testing, and to stay well hydrated. However, this was not specifically tested for by the investigators of this study.

### Sensor placement

**Figure 2:**
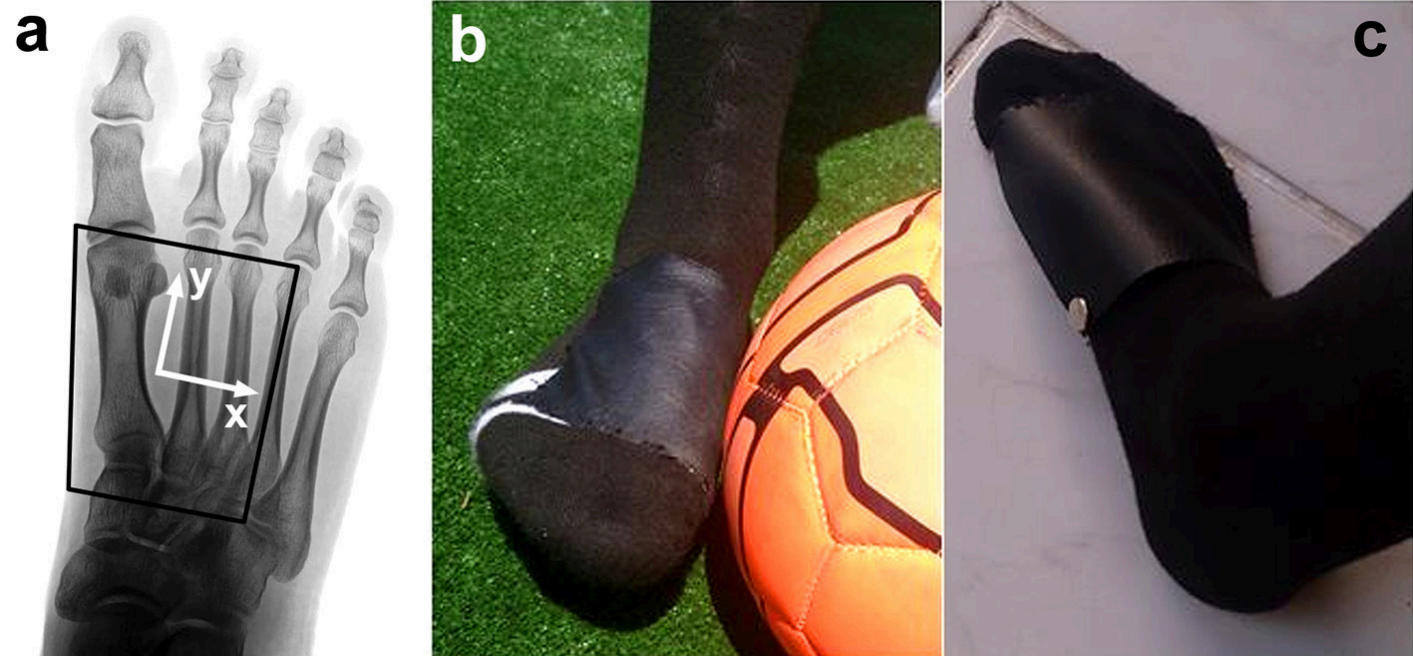
Sock with artificial leather stitched on top to secure the sensor matrix in place; **(a)** Placement of the sensor matrix (black contour; note that this black contour is not square as the sensor matrix is wrapped around the medial side of the foot, as seen in subfigure **c**), on anatomical landmarks and its coordinate system; **(b)** Instrumented sock and foot-to-ball contact; **(c)** Leather on top of the sensor matrix wrapped around the medial side of the foot, including snap fastener for securing the sensor matrix in place.

For the purpose of this study and for reasons of consistency and comparability, the sensor system had to be placed on specific anatomical landmarks to cover the contact area between the foot and the ball for the curved kicks. The sensor placement on the anatomical landmarks of the foot is visualized in Figure 2a. The sensor system is not implemented in a soccer boot yet and a design is warranted in which the sensor can be placed securely on the aforementioned anatomical landmarks.

To solve this issue, a placement of the sensor system inside a boot under the soccer shoes upper was tested. However, this approach was not satisfying since proper placement of the sensor cells could not be guaranteed and was rejected consequently.

Secondly, a pocket made of artificial leather was produced in which the sensor cells fit securely. This leather pocket could be fitted to the outer upper part of a soccer boot by Velcro tape. Even though being promising, this approach was rejected as well due to the same reasons as the first approach. The method of placing a pressure sensor on the shoe upper was actually used by Henning (Hennig et al., 2009; “*The pressure measuring pads were adjusted on top of the shoes to the foot anatomy, guaranteeing that all sensors were matched to identical anatomical locations of the individual feet*,” Hennig, 2011). However, we experienced problems of identifying the anatomical landmarks by palpation through the shoe upper, and therefore abandoned this method.

In a third approach, an off-the-shelf sock (EU-size 42–44) with a hand-stitched thin layer of artificial leather on top was used to build a pocket in which the sensor cells fit properly (Figures 2b,c). Artificial leather was chosen to mimic the upper material of commercially available soccer shoes as close as possible.

A snap fastener on one corner of the leather pocket allowed to insert and remove the sensor cells easily to allow maintenance if necessary. With this set up it is possible to equip different players easily with the sensor system, while simultaneously keeping the comfort of players high amidst kicking. Additionally, the design of the sock allowed a precise placement of the sensor system on the same anatomical landmarks for each participant, which is crucial for the purposes of this study. For these reasons, this approach was selected to analyses the characteristics of curved kicks with participants.

Sensor cell 1 was placed on the medial side of the metatarsophalangeal joint I. The medial edge of the sensor was aligned to the medial side of the metatarsal I in proximal direction to the medial cuneiform. The anterolateral corner of the sensor was located on the metatarsophalangeal joint IV. The lateral side of the sensor matrix was aligned to metatarsal IV.

### Experiments

To test the hypotheses, each player conducted 8–18 curved direct free kicks in windless conditions on artificial grass with a standard size 5 ball with an internal pressure of 0.8 bar (~11.6 psi). Players performed a standardized warm-up and were allowed to take several test kicks to familiarize themselves with the task prior to the actual testing. For all kicks, players were told to kick the ball as they would normally do in competition and not to alter their kicking technique in any way.

**Figure 3:**
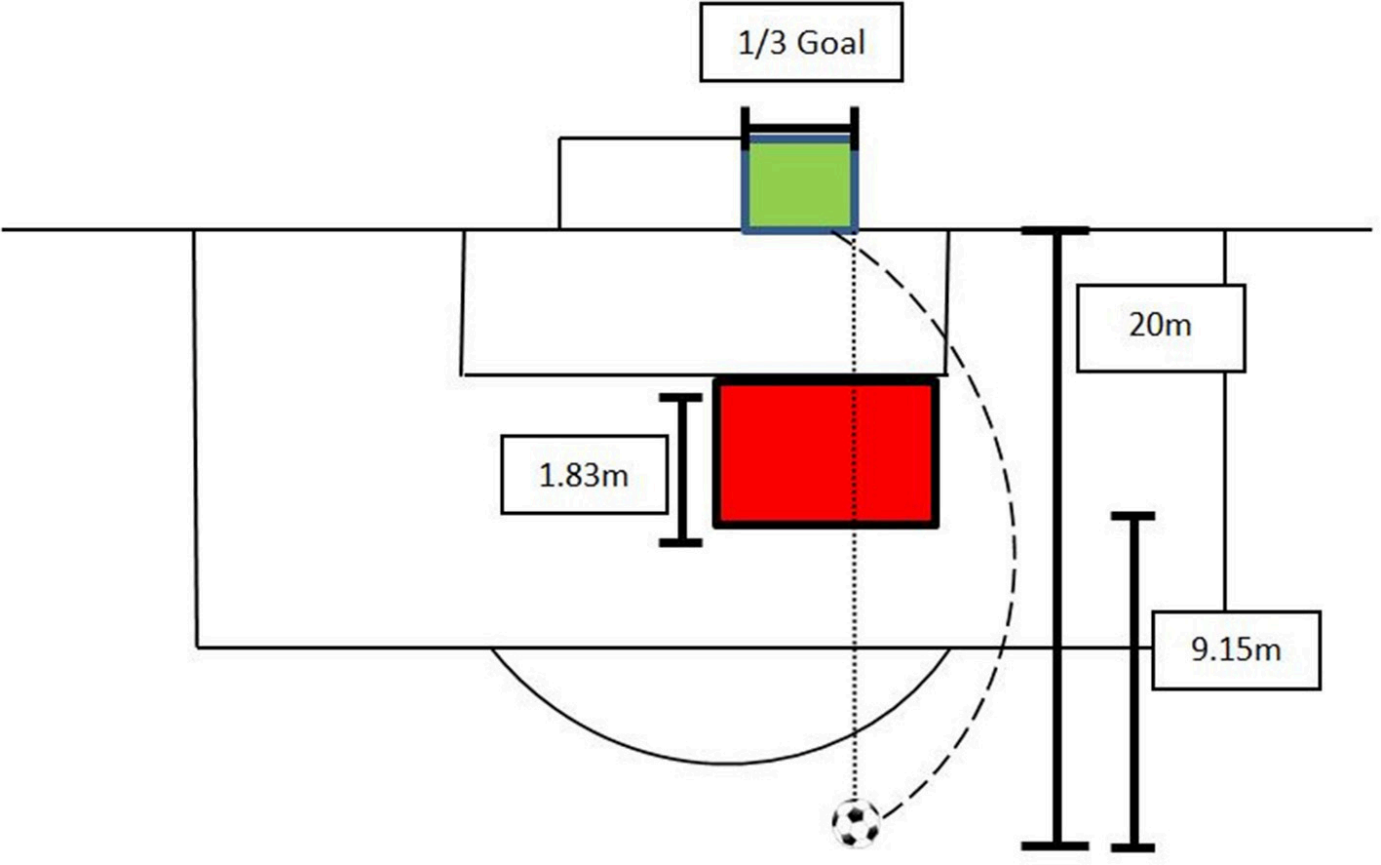
Schematic experimental set-up for the curved kick; players had to kick a left-curved kick around an artificial wall (red) inside the target (green); the dashed line represents the trajectory of the ball; the dotted line highlights that a straight shot will not deliver a successful kick.

Slightly modified from a previously used set-up (Alcock et al., 2012), the ball was positioned at 20 m distance in a straight line from the right goal post (Figure 3). An artificial wall made out of polymer material with a height of 1.83 m was placed at a distance of 9.15 m away from the ball and was placed sideways by an experienced goalkeeper as he/she would do in competition, i.e., $\frac{11}{2}$ players are placed outside of an imaginary line between the ball and the goal post closer to the ball. The aim for each player was to curve the ball around the artificial wall on the right side, and to hit a target with the dimensions of 2.44 × 2.44 m (1/3 of a full-sized goal) which was placed on the right side of the goal. Consequently, the ball had to follow a left-curved trajectory in order to hit the target. The kick was recorded as unsuccessful (miss) if the ball did not hit the target or was not curved around the artificial wall properly. Missed kicks were found to be on the right side of the target area, but never on the left side.

### Data analysis

The raw data of all 16 pressure sensor cells were collected at 2–2.5 kHz in ASCII format (10-bit analog to digital converter). The time series of the ASCII data was converted to voltage (drop voltage measured across the reference resistor of each of the 16 voltage dividers). From the voltage, the following parameters were calculated in sequence as a time series: the resistance of each pressure sensor (calculated from the voltage and the reference resistor); the conductance of each sensor (reciprocal of resistance); the pressure of each sensor (from the pressure-conductance-calibration curves); and the force on each sensor (from the sensor area and pressure). The overall impact force was determined from the sum of forces from the 16 individual cells. The center of pressure (COP) was calculated from individual forces and the position of the geometrical center of each sensor cell relative to the coordinate system of the sensor array (Figure 1) in x- and y-directions (COPx, COPy). The time derivatives of the distance between two consecutive COP positions delivered the instantaneous velocity of the COP.

We used the following continuous data as time series for further calculations: COPx, COPy, and impact force (F). The parameters used for statistical purposes were:

- COPx at maximal force;
- COPy at maximal force;
- Maximal impact force (Fmax).

All three parameters (quantitative data) were combined with the success data (qualitative binary data: hit = 1, miss = 0), the number of the participant and the number of kick. The latter two numbers served only for identification purposes, used for attributing parameter data to the participant and the type of kick. The success data served for calculation of the probability of success *P*, of scoring a goal.

#### Hits against misses analysis

The data of the parameters (listed above) of all hits were compared to the parameters of all misses with the Mann–Whitney *U*-test (as some of the data sets were not normally distributed, verified with the Shapiro–Wilk test if *p* < 0.05) and the *p*-values were determined. This procedure revealed whether there is a significant difference between parameter data obtained from kicking a successful or unsuccessful curve shot. The effect size was calculated in terms of the Rank-Biserial Correlation (Cureton, 1956), *r*, from the *U*-value: *r* = 1 – 2*U*/(*n*_1_ *n*_2_), where *n*_1_ and *n*_2_ denote the number of data compared by the Mann–Whitney test, and *U* ≤ 0.5 *n*_1_ *n*_2_. Note that the effect size *r* ranges from zero to unity.

#### Regression analysis

The probability of success *P*, of scoring a goal, equals the average of hits *h* (1) and misses *m* (0) across a specified parameter range.

(1) $$\begin{array}{r} P=\frac{\sum \left(h,m\right)}{n} \end{array}$$

where *n* is the total number of data.

The method used in this paper is an *analogy* to, and *optimization* of, the *Median–Median Line* method by Wald (1940). However, instead of dividing the data into two *equal* size subsamples, separated by the median of the independent parameter, the separation line divided the data sample into two *unequal* size subsamples, which was optimized based on the conditions explained subsequently.

The entire dataset of an independent parameter including the associated hit and miss data (dependent parameter), was divided into two subsamples (data ranges), separated by a threshold value *s*. The subsample on one side of *s* delivers a greater probability of success *P*, compared to the subsample on the other side of *s*. The preferred range, for maximizing the chances of success, is identified by the higher *P*. The absolute *P*-differential *D* of the two subsamples should be as high as possible.

(2) $$\begin{array}{r} D=P_{1}-P_{2}=\frac{\overset{i_{s}}{\underset{i=1}{\sum }}\left(h,m\right)}{i_{s}}-\frac{\overset{n}{\underset{i=i_{s}}{\sum }}\left(h,m\right)}{n-i_{s}} \end{array}$$

where *i*_*s*_ denotes the number of the datum just before or after the threshold value *s*; *P*_1_ denotes *P* before *s*, and *P*_2_ denotes *P* after *s*; by definition, the average *P*_1_ is greater than the average *P*_2_, in order to fulfill the condition of a maximum or near-maximum *D*.

Yet, the probability data *P*, on either side of *s*, should be significantly different. This was determined with an independent *t*-test, by comparing the two samples of hit and miss data (*h, m*) of both sides of *s*. An *F*-test for testing the significance of the difference between the variances of the two samples determined whether a homoscedastic (*F*-test *p* > 0.05) or heteroscedastic (*F*-test *p* < 0.05) *t*-test had to be performed. These homo- and hetero-scedastic *p*-values as well as the *F*-test *p*-value were computed with a moving average (smaller and greater *s*) across the entire dataset, i.e., for all possible *s*-values running across the entire range of a parameter (such as COPy, Fmax, etc.). The optimal threshold value *s* was determined from those *D*-data that are

1. close to, or at, the maximum *D*,
2. exhibit a *t*-test *p* < 0.05, and
3. have at least 20% of the data on either side of *s*.

**Figure 4:**
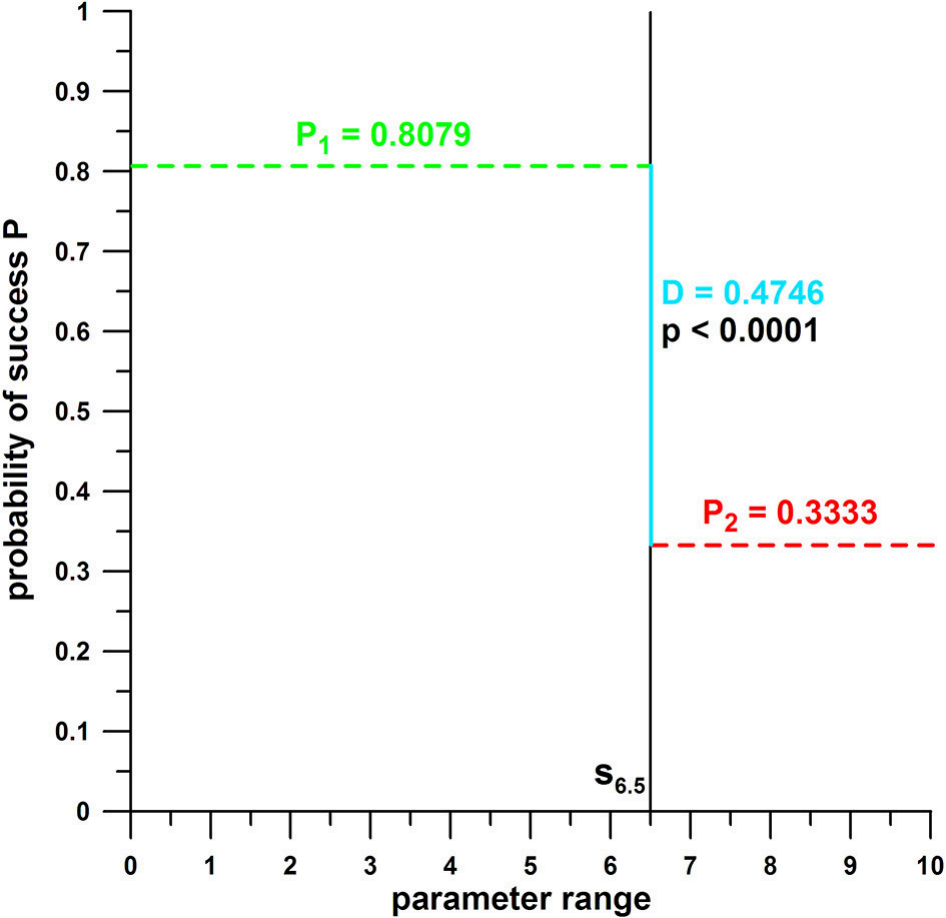
Principle of probability of success (P) against the range of parameter data; the black vertical line *s* divides the parameter range into two subsamples (smaller and greater than *s*); P_1_ = average of hit and miss data for the parameter range smaller than the threshold value *s* (s = 6.5); P_2_ = average of hit and miss data for the parameter range larger than the threshold value *s*; D = probability differential (P_1_ – P_2_); P_1_ and P_2_ are significantly different (*p* = *p*-value); the parameter range associated with *P*_1_, the larger of the two *P*, is the favorable range of the parameter tested; the parameter range associated with *P*_2_, the smaller of the two *P*s, is the *un*favorable range of the parameter tested.

**Figure 5:**
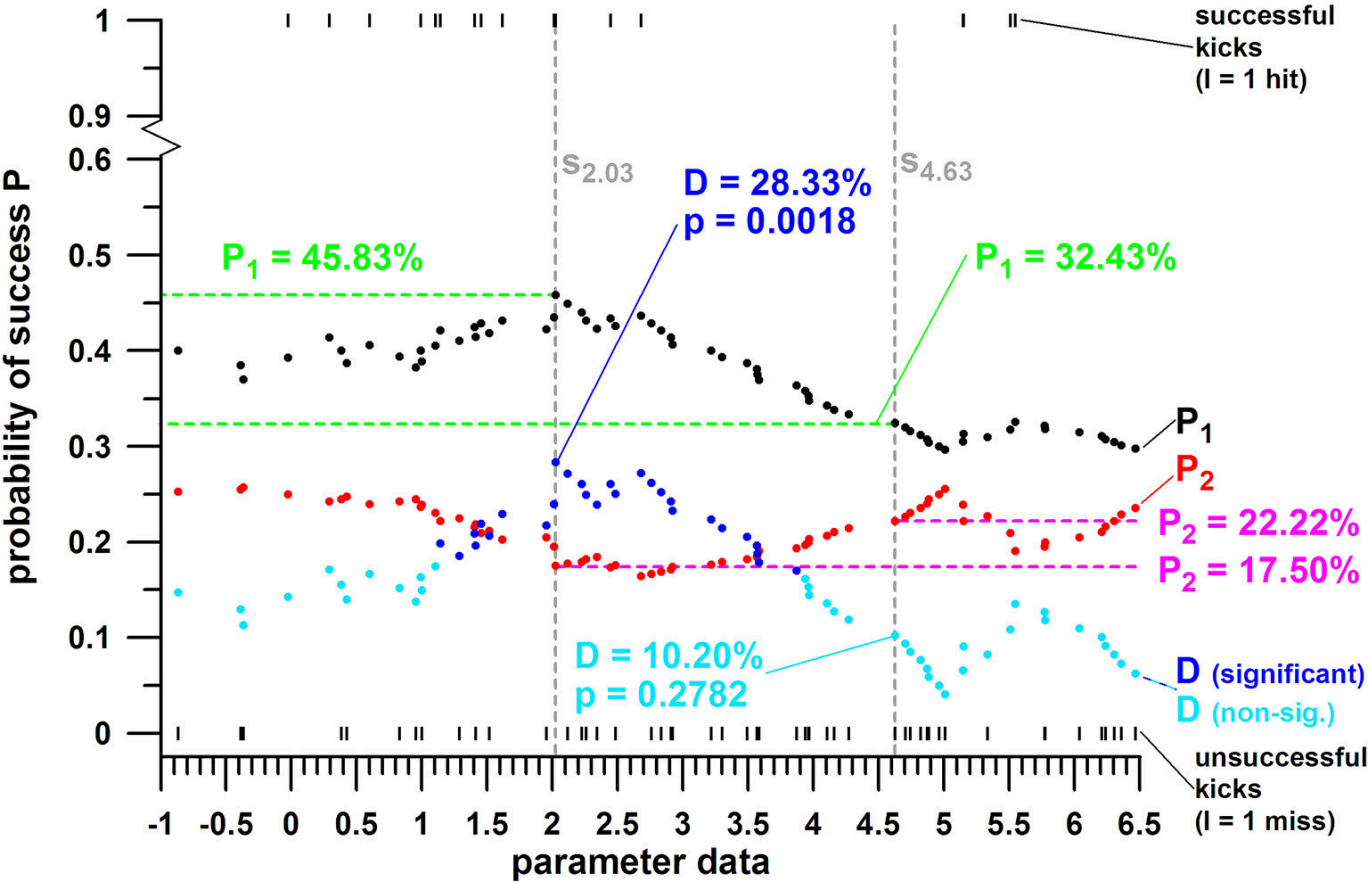
Example of probability of success (P) against the range of parameter data with two threshold value *s* (*s* = 2.03 and *s* = 4.63); P_1_ = average of hit and miss data for the parameter range smaller than the threshold value *s* (s = 6.5); P_2_ = average of hit and miss data for the parameter range larger than the threshold value *s*; D = probability differential (P_1_ – P_2_; D is greater for *s* = 2.03); P_1_ and P_2_ are significantly different (*p* = *p*-value) for *s* = 2.03 and insignificantly different for *s* = 4.63.

The last requirement ensures that there is a sufficient number of data left for the Kruskal–Wallis rank sum test, detailed in the next section. The optimal threshold value *s* divides the parameter range into two subsamples (data ranges), a favorable one (for maximizing the chances of success) and an unfavorable one (that minimizes the chances of success Figure 4). When comparing the data of the two subsamples, the effect size is always at the maximum, as they are separated by *s*. Figure 5 is an extension of Figure 4, showing a realistic dataset and a feasible (ideal) and an unfeasible separation line *s*. The feasibility is determined by the *p*-value and the magnitude of *D*.

#### Two-parameter analysis

In contrast to the previous section that treats each parameter individually, this section deals with the effect of two parameters have on each other, i.e., addresses the question whether the favorable ranges of two parameters influence each other positively (by improving the probability of scoring a goal) or negatively (by diminishing the probability of scoring a goal). When selecting two parameters, then, based on their individual threshold values, four combinations (quarters of a point cloud), and associated datasets of hit and miss data, are obtained:

- parameter A, *un*favorable data range, and parameter B, *un*favorable data range;
- parameter A, favorable data range, and parameter B, *un*favorable data range;
- parameter A, *un*favorable data range, and parameter B, favorable data range;
- parameter A, favorable data range, and parameter B, favorable data range.

The four associated datasets of hit and miss data, resulting in four average probability (*P*) data, were tested for their significant differences. It was expected that the probability of success (*P*) of two parameters combined, both in their favorable ranges,

- was greater than *P* of either of these parameters individually in their favorable ranges; and
- was *significantly* greater than P of two parameters combined, both in their *un*favorable ranges.

The significance of the latter expectation was tested with Kruskal–Wallis rank sum test, and the significance of the individual differences in the four average probability (*P*) data was assessed with two *post-hoc* tests: Conover and Dunn, both of them adjusted by the Holm FWER (familywise error rates) and Benjamini-Hochberg FDR (false discovery rate) methods. The effect size was calculated in terms of the Rank-Biserial Correlation *r*, by comparing the data sets of the two parameters in their favorable and *un*favorable ranges. It is evident that the effect size results in 1 for parameter A and B (when comparing data of the favorable and *un*favorable ranges); however, the third parameter (C) is not optimized (in terms of favorable and *un*favorable ranges), the effect size of which is therefore smaller than unity.

#### Three-parameter analysis

When selecting three parameters, then, based on their individual threshold values, eight combinations and associated datasets of hit and miss data are obtained:

- parameter A, *un*favorable data range; parameter B, *un*favorable data range; and parameter C, *un*favorable data range;
- parameter A, *un*favorable data range; parameter B, *un*favorable data range; and parameter C, favorable data range;
- parameter A, favorable data range; parameter B, *un*favorable data range; and parameter C, *un*favorable data range;
- parameter A, favorable data range; parameter B, *un*favorable data range; and parameter C, favorable data range;
- parameter A, *un*favorable data range; parameter B, favorable data range; and parameter C, *un*favorable data range;
- parameter A, *un*favorable data range; parameter B, favorable data range; and parameter C, favorable data range;
- parameter A, favorable data range; parameter B, favorable data range; and parameter C, *un*favorable data range;
- parameter A, favorable data range; parameter B, favorable data range; and parameter C, favorable data range;

The eight associated datasets of hit and miss data, resulting in eight average probability (*P*) data, were tested for their significant differences. It was expected that the probability of success (*P*) of three parameters combined, both in their favorable ranges, was significantly greater than *P* of two parameters combined, both in their *un*favorable ranges.

The significance of the latter expectation was tested with Kruskal–Wallis rank sum test, and the significance of the individual differences in the average probability (*P*) data was assessed with the *post-hoc* tests specified above.

In the three-parameter analysis, the conditions for finding the optimal threshold value *s* were re-defined such that:

1. The associated *D*-data exhibit a *t*-test *p* < 0.05;
2. The new threshold value *s* have at least 20% of the data on either side of *s*;
3. The chance of success of scoring a goal, for all three parameters in their favorable ranges, is at the maximum; and
4. The chances of success of scoring a goal, for parameter combinations with at least one of the parameters in its unfavorable range, are significantly different from the chance of success for all three parameters in their favorable ranges, to be verified by the *post-hoc* tests applied as specified above.

Note that one of the previous conditions of the regression analysis (single parameter), namely *s* close to, or at, the maximum *D*, was sacrificed for obtaining the highest chance of success of scoring a goal with all three parameters in their favorable ranges. The effect size (Rank-Biserial Correlation *r*) is always unity when comparing the data sets of the three parameters in their favorable and *un*favorable ranges.

#### COP analysis

In order to establish a difference in the COP path of successful and unsuccessful kicks, the average paths of COPx and COPy, and the average impact forces at each COP position, were calculated from the two-parameter analyses, by taking the successful kicks of the two parameters in the favorable range, and the *un*successful kicks of the two parameters in the *un*favorable range. By taking several combinations of two parameters, the COP paths of the successful kicks as well as the ones of the *un*successful ones should be close to each other and thereby mutually validate the sweet spot on the boot. The COP path was visualized as a bubble plot, where the bubble size corresponded to the magnitude of the impact force.

The COPs (and also Fmax) of similar kicks (successful or *un*successful) were averaged in the following way:

- COPx at Fmax, COPy at Fmax, and Fmax were aligned such that they shared the same data sequence number (or time stamp);
- as the number of data before and after the “peak datum number” was unequal across the kick datasets, the peak data (COPx at Fmax; COPy at Fmax; Fmax) were averaged first across all successful kicks and then across all *un*successful kicks;
- subsequently, the datasets were adjusted such that they shared the same average peak data; this was required for averaging the data tails before and after the “peak datum number”; for example, when comparing two kicks, a kick with a more proximal COPy and a shorter tail, and a kick with a more distal COPy and a longer tail will certainly result in an average COPy located halfway between the overlapping segments of the two tails; in contrast, the excess data of the longer tail outside the overlapping segment would remain on the distal side without representing any average; thus, the adjustment is needed to avoid this issue;
- finally, the datasets were averaged across all data sequence numbers; for small and high data sequence numbers (i.e., at the tails of the individual datasets), the number of data that were averaged was smaller than for the data averaged at the peak datum number; averages that were based on less than a third of the number of data averaged at the peak datum number were discarded, as they did no longer represent the group average;
- the average data were plotted against the data sequence numbers, and a polynomial function of a higher order was fitted to the data (COPx, COPy, F). The optimal polynomial order was determined with a convergence test at which the *R*^2^-value (coefficient of determination) of the fit started to asymptote;
- the fit function was used to produce a smooth COP and force data sequence, displayed as a bubble plot. The varying bubble size corresponded to the simultaneous impact force at the individual bubble.

## Results

The Results section is organized around the four main findings in consecutive order whereby one finding leads to the next one:

1. the data comparison of all hits and all misses proved unsuccessful for establishing sweet and dead spots;
2. the trend analysis confirmed favorable and *un*favorable parameter ranges for COPx, COPy, and Fmax;
3. the chances of scoring a goal were significantly higher if two or all three parameters are in their favorable ranges (i.e., at the sweet spot with chances of 58–86%) compared to two or all three parameters in their unfavorable ranges (i.e., at the dead spot with chances of 11–22%);
4. the sweet spot locations obtained from two- to three-parameter analyses were identical, but clearly separated from, and located more medial and proximal than, the more scattered dead spots.

### Participant statistics

The participants kicked the ball 8–18 times (12.9 ± 3.1). Their chances of success of scoring a goal ranged from 22.2 to 72.7% (30.3 ± 20.3%). Only two of the 10 players scored in more than 50% of the attempts.

### Comparison of parameter data of all hits against all misses

The peak force (Fmax) data of all misses and all hits were 1,682 ± 519 N (678–3,161), and 1,843 ± 628 N (769–3,365), respectively. The COPx data (at Fmax) were −7.9 ± 8.0 mm (−24.3 to +20.1 mm) and −10.2 ± 7.4 mm (−22.9 to +12.4 mm), respectively; and COPy data (at Fmax) were 3.7 ± 4.4 mm (−10.4 to +15.1 mm) and 3.0 ± 5.4 mm (−4.4 to +17.2 mm).

The *p*-values of the three parameters were >0.05 and therefore the parameter data of all hits were not different from the parameter data of all misses. Specifically, the *p*-value of COPx of all hits compared to COPx of all misses was 0.187; the corresponding *p*-value of COPy was 0.105; and the one of Fmax was 0.119. As there was no difference between parameters of hits and misses, only a *very small* (if 0.01 < *r* < 0.2; Sawilowsky, 2009) effect was observed: COPx effect size *r* = 0.149; COPy effect size *r* = 0.183; and Fmax effect size *r* = 0.176. Hypothesis 1 was therefore confirmed and the method of comparing the parameter data of all hits against all misses is considered unsuccessful.

### Trend analysis

For the three parameters defined in the Methodology section, the threshold values *s, P*_1_ before and *P*_2_ after the threshold, the probability differential *D* at the threshold, the *p*-value of *D*, the number of significant data, and the overall trend are listed in Table 1.

**Table 1:** Summary of trend analyses; *s* = parameter value at the separation line, separating the favorable parameter range from the unfavorable one; *D* = differential of *P*_1_ (probability of scoring a goal if the parameter is in the favorable range) and *P*_2_ (probability of scoring a goal if the parameter is in the unfavorable range); COPx, center of pressure in x-direction; COPy, center of pressure in y-direction; Fmax, maximum impact force.

	**COPx1**	**COPx2**	**COPx3**	**COPy**	**Fmax**
*s*	−0.0086 m	−0.007 m	−0.0036 m	0.0021 m	2105 N
*D* at *s*	0.1676	0.1630	0.1871	0.2833	0.2422
*p*-value of *D*	0.032	0.040	0.020	0.002	0.005
no. of significant *D*-data (having at least 20% of the data on either side of *s*)	2	1	2	29	15
*P*_1_	0.3676	0.3553	0.3300	0.4583	0.4667
*P*_2_	0.2000	0.1923	0.1429	0.1750	0.2245
Trend	The smaller (= more on the *medial* side), the better	The smaller (= more on the *medial* side), the better	The smaller (= more on the *medial* side), the better	The smaller (= more on the *proximal* side), the better	The higher, the better

**Figure 6:**
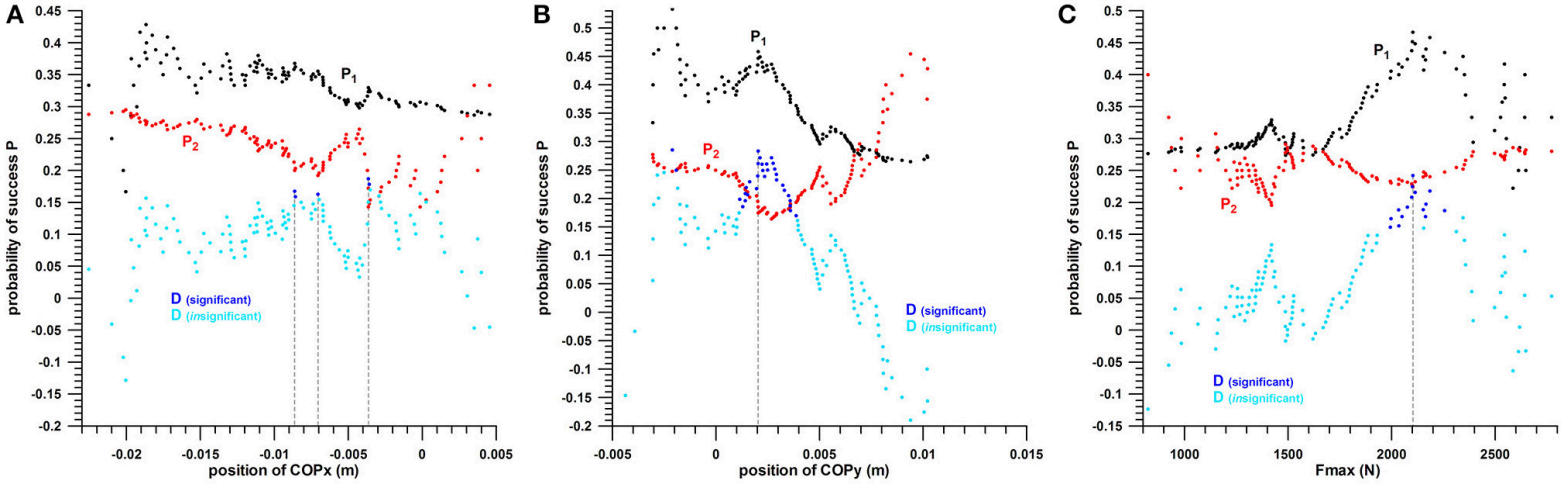
Probability P of success of scoring a goal against the parameter range; P_1_ = probability of scoring a goal if the parameter is in the favorable range; P_2_ = probability of scoring a goal if the parameter is in the unfavorable range; D = differential of P_1_ and P_2_; **(A)**: P against COPx (location of the COP in x-direction); **(B)**: P against COPy (location of the COP in y-direction); **(C)**: P against Fmax (peak impact force); note that D can be negative, if P_2_ > P_1_.

COPx exhibited three possible threshold values (Figure 6A), the highest one with the best *D, p*-value, and *P*_2_; and the smallest one with the best *P*_1_. In the two remaining parameters (Figures 6B,C), there was only one threshold value that satisfied the conditions of

1. having the maximum *D*,
2. exhibiting a *t*-test *p* < 0.05, and
3. having at least 20% of the data on either side of *D*_max_.

*P*_1_ ranged from 33 to 47% (the higher, the better); *P*_2_ from 14 to 22% (the smaller, the better); and *D* from 16 to 28% (the higher, the better).

As favorable and unfavorable parameter ranges could be found, Hypothesis 2 was confirmed.

### Two-parameter analysis

The hit/miss data were divided into 4 groups for pairwise comparison (Table 2), and the chances of success of the groups were compared with the Kruskal–Wallis rank sum test.

**Table 2:** Combinations of two parameters and their group code used in Figures 7–9, as well as in Figure 10 (combination of three parameters); COPx, center of pressure in x-direction; COPy, center of pressure in y-direction; Fmax, maximum impact force.

**Group code**	**COPx at Fmax vs. COPy at Fmax**	**COPx vs. Fmax**	**COPy vs. Fmax**
I	Position of COPx within the *un*favorable range, and position of COPy within the *un*favorable range	Position of COPx within the *un*favorable range, and magnitude of Fmax within the *un*favorable range	Position of COPy within the *un*favorable range, and magnitude of Fmax within the *un*favorable range
II	Position of COPx within the *un*favorable range, and position of COPy within the favorable range	Position of COPx within the *un*favorable range, and magnitude of Fmax within the favorable range	Position of COPy within the *un*favorable range, and magnitude of Fmax within the favorable range
III	Position of COPx within the favorable range, and position of COPy within the *un*favorable range	Position of COPx within the favorable range, and magnitude of Fmax within the *un*favorable range	Position of COPy within the favorable range, and magnitude of Fmax within the *un*favorable range
IV	Position of COPx within the favorable range, and position of COPy within the favorable range	Position of COPx within the favorable range, and magnitude of Fmax within the favorable range	Position of COPy within the favorable range, and magnitude of Fmax within the favorable range

#### COPx at Fmax vs. COPy at Fmax

**Figure 7:**
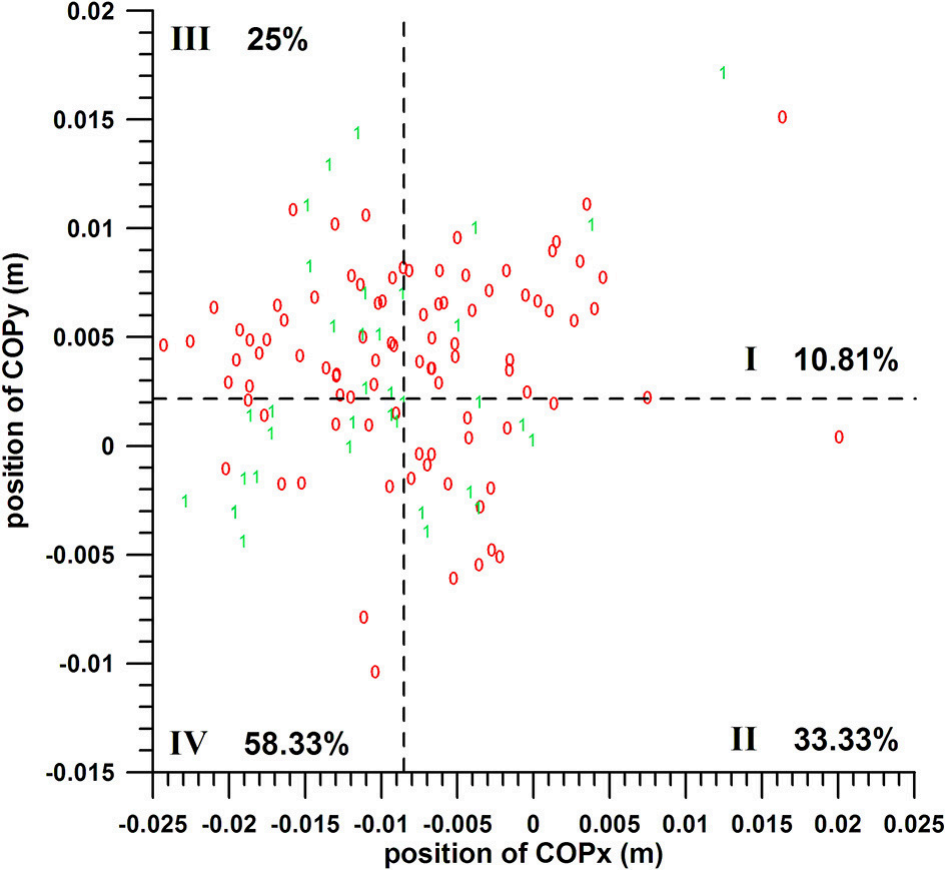
Two-parameter analysis: COPx vs. COPy; 0 = miss, 1 = hit; dashed lines = threshold values (cf. Table 1); I, II, III, IV = group codes from Table 2.

The chances of success of the groups I, II, III, and IV (Table 2) were:

COPx threshold value *s* at −0.0086 m: 10.81, 33.33, 25, and 58.33%, respectively (quarter analysis from Figure 7);

COPx threshold value *s* at −0.0070 m: 12.12, 30, 22.92, and 57.14%, respectively;

COPx threshold value *s* at −0.0036 m: 10.53, 20, 20.97, and 52.63%, respectively.

The difference between these group percentages of success was statistically highly significant as determined by the Kruskal–Wallis rank sum test (*p* < 0.0013 for the three COPx threshold values). The *post-hoc* tests revealed the individual differences, namely that the percentage of a parameter of group IV (>50%) was significantly different from the percentages of groups I and III; whereas the remaining pairs were not significantly different, among which is II vs. IV, for all the three COPx threshold values.

The individual percentages of the success probability of COPx and COPy positions (both in their favorable ranges) were 33–36.8 and 45.80%, respectively; their combined success probability exceeded the individual ones and was 52.6–58.3%.

#### COPx vs. Fmax

**Figure 8:**
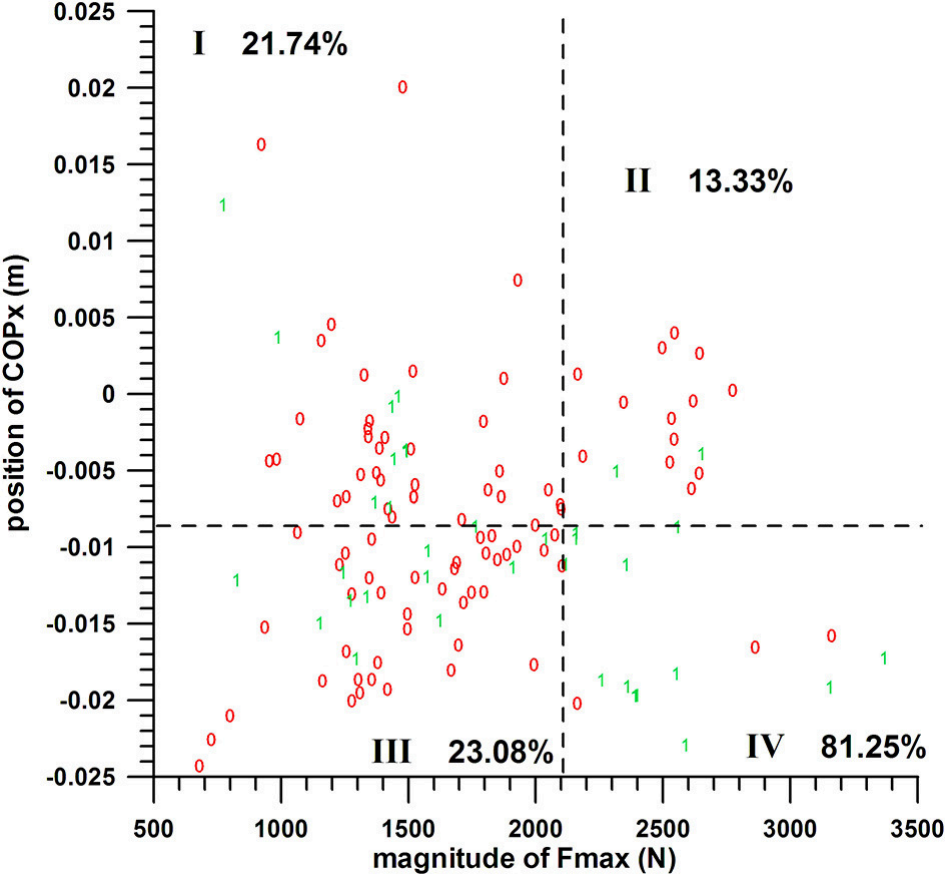
Two-parameter analysis: COPx vs. Fmax (peak impact force); 0 = miss, 1 = hit; dashed lines = threshold values (cf. Table 1); I, II, III, IV = group codes from Table 2.

The chances of success of the groups I, II, III, and IV were:

COPx threshold value s at −0.0086 m: 21.74, 13.33, 23.08, and 81.25%, respectively (quarter analysis from Figure 8);

COPx threshold value s at −0.007 m: 21.05, 13.33, 23.33, and 81.25%, respectively;

COPx threshold value s at −0.0036 m: 20, 0, 23.08, and 68.18%, respectively.

The difference between these group percentages of success was statistically highly significant as determined by the Kruskal–Wallis rank sum test (*p* ≤ 0.00006 for the three COPx threshold values). The *post-hoc* tests revealed the individual differences, namely that the percentage of group IV (>68%) was significantly different from the three other percentages; whereas the other three were not significantly different.

The individual percentages of the success probability of the COPx position and Fmax magnitude (both in their favorable ranges) were 33–36.8 and 46.67%, respectively; their combined success probability exceeded the individual ones and was >68%.

#### COPy vs. Fmax

**Figure 9:**
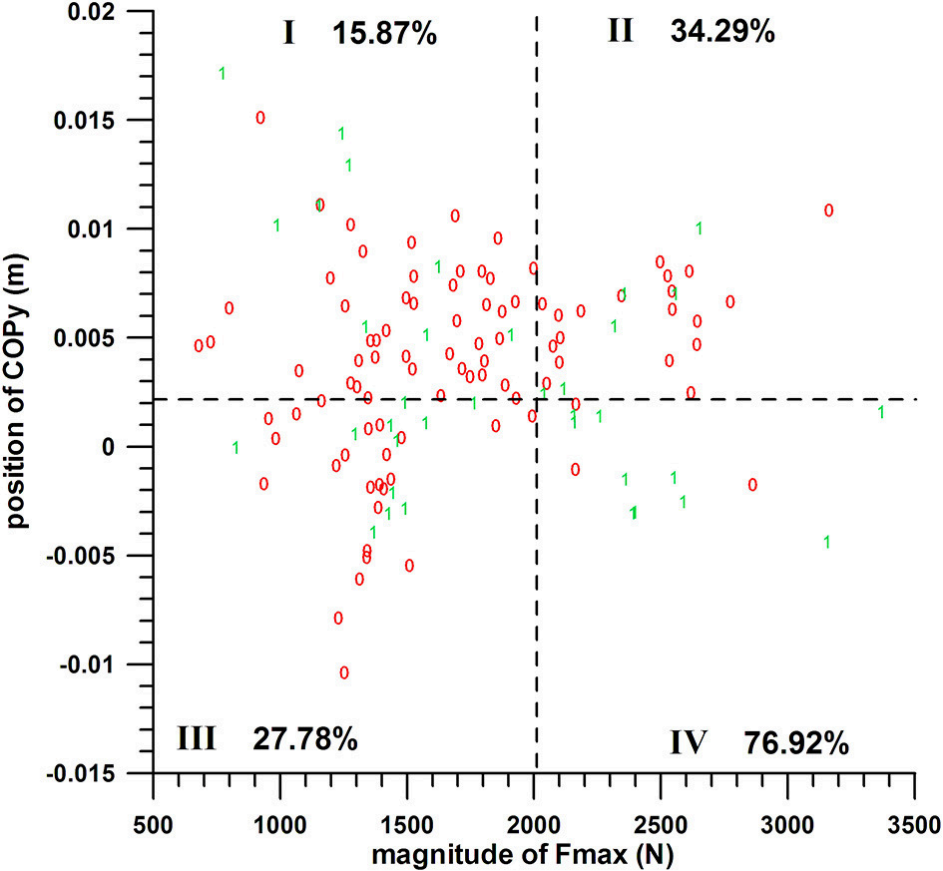
Two-parameter analysis: COPy vs. Fmax (peak impact force); 0 = miss, 1 = hit; dashed lines = threshold values (cf. Table 1); I, II, III, IV = group codes from Table 2.

The chances of success of the groups A, B, C, and D were 15.87, 34.29, 27.78, and 76.92%, respectively (quarter analysis from Figure 9). The difference between these group percentages of success was statistically highly significant as determined by the Kruskal–Wallis rank sum test (*p* = 0.000152). The *post-hoc* tests revealed the individual differences, namely that the percentage of parameter D (77%) was significantly different (*p* < 0.015) from the three other percentages; whereas the other three were not significantly different (*p* > 0.06).

The individual percentages of the success probability of the COPy position and Fmax magnitude (both in their favorable ranges) were 45.83 and 46.67%, respectively; their combined success probability exceeded the individual ones and was 77%.

### Three-parameter analysis

The hit/miss data were divided into eight groups initially for pairwise comparison:

Ia: position of COPx within the unfavorable range, position of COPy within the unfavorable range, and magnitude of Fmax within the unfavorable range
IIb: position of COPx within the unfavorable range, position of COPy within the unfavorable range, and magnitude of Fmax within the favorable range
IIa: position of COPx within the unfavorable range, position of COPy within the favorable range, and magnitude of Fmax within the unfavorable range
IIb: position of COPx within the unfavorable range, position of COPy within the favorable range, and magnitude of Fmax within the favorable range
IIIa: position of COPx within the favorable range, position of COPy within the unfavorable range, and magnitude of Fmax within the unfavorable range
IIIb: position of COPx within the favorable range, position of COPy within the unfavorable range, and magnitude of Fmax within the favorable range
IVa: position of COPx within the favorable range, position of COPy within the favorable range, and magnitude of Fmax within the unfavorable range
IVb: position of COPx within the favorable range, position of COPy within the favorable range, and magnitude of Fmax within the favorable range

**Figure 10:**
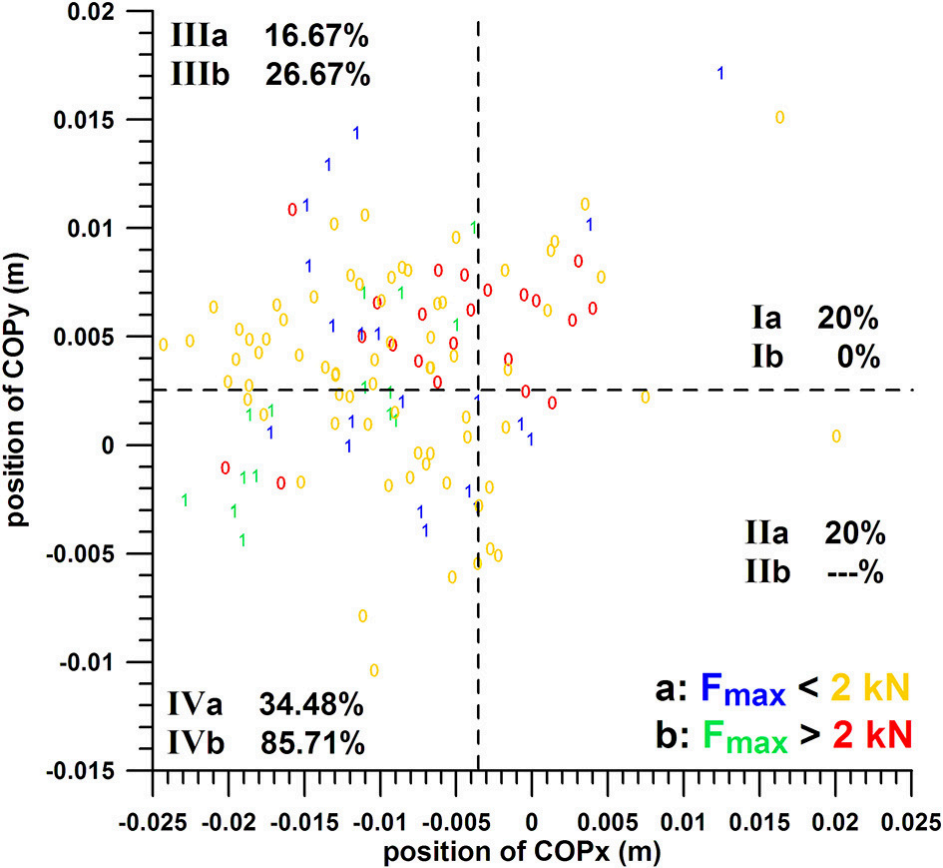
Three-parameter analysis: positions of COPy against COPx; 1 = hit, 0 = miss; the dashed lines separate the favorable from the unfavorable ranges of COPx and COPy; I, II, III, IV = group codes from Table 2, i.e., combinations of COPx and COPy; suffixes a and b denote Fmax in the unfavorable (Fmax < 2 kN) and favorable ranges (Fmax > 2 kN), respectively; Ia = parameter combination with COPx, COPy, and Fmax in their unfavorable ranges; IVb = all three parameters in their favorable range.

Note that the suffixes “a” and “b” refer to Fmax within the unfavorable and favorable ranges, respectively. Group IIb was excluded as it consisted only of 2 data (both were misses; cube analysis from Figure 10, red zeros in quadrant II). The chances of success of the groups were compared with the Kruskal–Wallis rank sum test.

The threshold values for separating favorable and unfavorable ranges were re-defined in order to obtain the highest chance of success (percentage) of group IVb. The separation values for max percentage of success (85.71%) were found for COPx at −0.0036 m, COPy at 0.0027 m, and for Fmax at 2,000 N; thereby satisfying the conditions stated in the Methodology section of the three-parameter analysis. At these separation values, the chances of success for scoring a goal with two parameters in their favorable ranges out of these three parameters were: COPx vs. COPy, 51.16%; COPx vs. Fmax, 55.17%; COPy vs. Fmax: 75%. These two-parameter percentages were smaller than the optimal ones found in the two-parameter analysis, namely COPx vs. COPy: 52.63–58.33% (three different s for COPx), COPx vs. Fmax: 68.18–81.25% (three different s for COPx), COPy vs. Fmax: 76.92%.

The chances of success of the groups Ia, Ib, IIa, IIIa, IIIb, IVa, and IVb were 20, 0, 20, 16.67, 20.67, 34.48, and 85.71%, respectively (cube analysis from Figure 10). The difference between these group percentages of success was statistically highly significant as determined by the Kruskal–Wallis rank sum test (*p* = 0.000067). The *post-hoc* tests revealed the individual differences, namely that the percentage of group IVb (85.71%, all three parameters in their favorable ranges) was significantly different from all the other groups (*p* < 0.01). In contrast to this, the percentage of the other groups (excluding IVb) were not significantly different.

The individual percentages of the success probability of COPx and COPy positions and Fmax magnitude (both in their favorable ranges) were 33–36.8, 45.83, and 46.67%; their combined success probability in the two-parameter analysis was 51.16–75% (see above); and their combined success probability in the three-parameter analysis arrived at 85.71%, which exceeded the individual and two-parameter combination ones.

### Path of the COP

**Figure 11:**
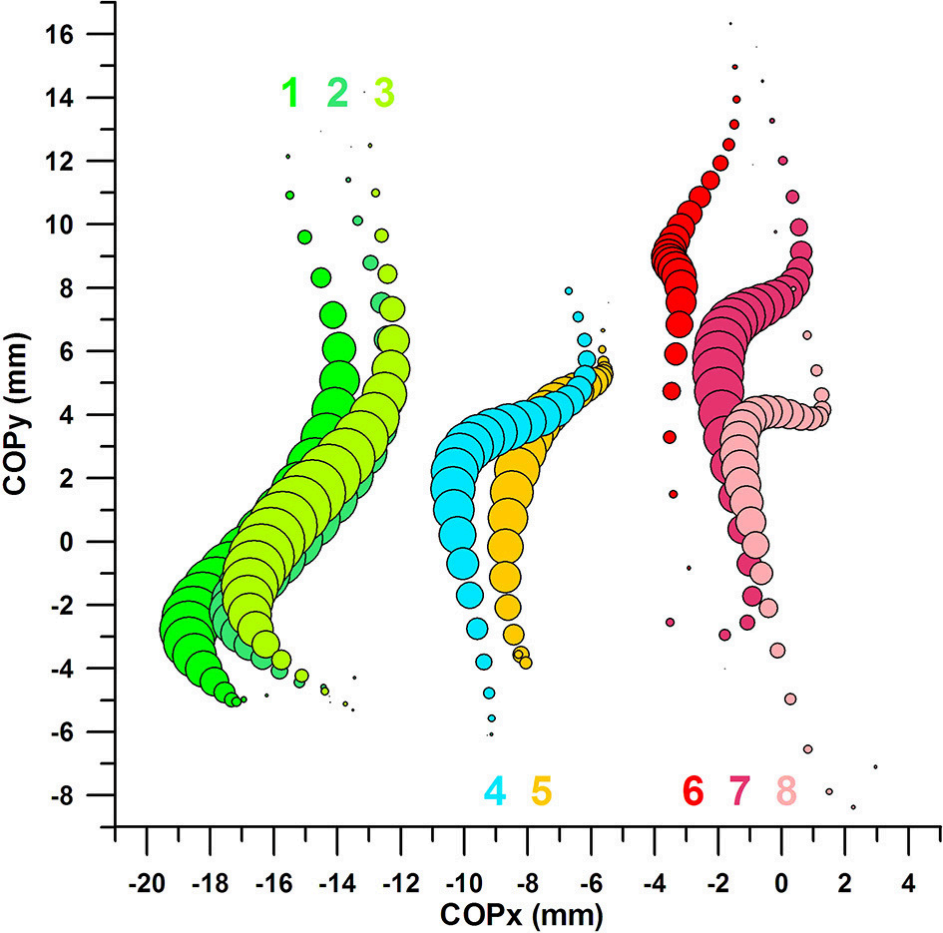
Centre of pressure in y-direction (COPy) against Centre of pressure in x-direction (COPx); the bubble size of the 8 bubble plots corresponds to the impact force; the graphs are aligned to the coordinate system of the sensor matrix: positive COPy data = distal; negative COPy data = proximal; negative COPx data = medial; positive COPx data = lateral; the COP moves from distal to proximal (= downward on the graph) during impact; 1: average COP of successful kicks, COPy and Fmax within the favorable range; 2: average COP of successful kicks, COPx and COPy within the favorable range; 3: average COP of successful kicks, COPx and Fmax within the favorable range; 4: average COP of all successful kicks; 5: average COP of all *un*successful kicks; 6: average COP of *un*successful kicks, COPy and Fmax within the *un*favorable range; 7: average COP of *un*successful kicks, COPx and COPy within the *un*favorable range; 8: average COP of *un*successful kicks, COPx and Fmax within the *un*favorable range.

Figure 11 shows eight datasets, numbered from 1 to 8 from medial (left) to lateral (right):

- dataset 1: average COP of successful kicks; COPy and Fmax within the favorable range
- dataset 2: average COP of successful kicks; COPx and COPy within the favorable range
- dataset 3: average COP of successful kicks; COPx and Fmax within the favorable range
- dataset 4: average COP of all successful kicks
- dataset 5: average COP of all *un*successful kicks
- dataset 6: average COP of *un*successful kicks; COPy and Fmax within the *un*favorable range
- dataset 7: average COP of *un*successful kicks; COPx and COPy within the *un*favorable range
- dataset 8: average COP of *un*successful kicks; COPx and Fmax within the *un*favorable range

#### COP of all successful kicks compared to COP of all unsuccessful kicks

The COP moves form distal to proximal, with a slight movement to the medial side (Figure 11, datasets 4 and 5). The COP of all successful kicks appears to be located more medially (at least after the force peak) compared to the COP of all *un*successful kicks; this apparent difference, however, is statistically not significant and therefore due to chance. From the Results section 2, COPx at Fmax, COPy at Fmax, and Fmax of all successful and *un*successful kicks are similar.

#### COP of successful kicks with COPx and COPy in their favorable ranges compared to COP of unsuccessful kicks with COPx and COPy in their unfavorable ranges

The average COP paths of successful (dataset 2, Figure 11) and *un*successful (dataset 7, Figure 11) kicks were clearly separated; the COPx data at Fmax (medio-laterally) by ~13 mm (Figure 11); and the COPy data at Fmax (proximo-distally) by ~6 mm. The peak forces of both datasets (2, 7) were identical (Mann–Whitney *U*-test *p* = 0.984; negligible effect size of *r* = 0.005).

#### COP of successful kicks with COPx and Fmax in their favorable ranges compared to COP of unsuccessful kicks with COPx and Fmax in their unfavorable ranges

The average COP paths of successful (dataset 3, Figure 11) and unsuccessful (dataset 8, Figure 11) kicks were clearly separated in the x-direction; however, there was no significant difference between the COPy at Fmax data of both datasets (Mann–Whitney *U*-test *p* = 0.2113; small effect size of *r* = 0.212).

#### COP of successful kicks with COPy and Fmax in their favorable ranges compared to COP of unsuccessful kicks with COPy and Fmax in their unfavorable ranges

The average COP paths of successful (dataset 1, Figure 11) and unsuccessful (dataset 6, Figure 11) kicks were clearly separated; surprisingly, there was a significant difference between the COPx at Fmax data of both datasets (Mann–Whitney *U*-test *p* = 0.0033; medium effect size of *r* = 0.521), even if the COPx data were not optimized in this analysis (only COPy and Fmax were).

#### COP of successful kicks with COPx, COPy, and Fmax in their favorable ranges compared to COP of unsuccessful kicks with COPx, COPy, and Fmax in their unfavorable ranges

**Figure 12:**
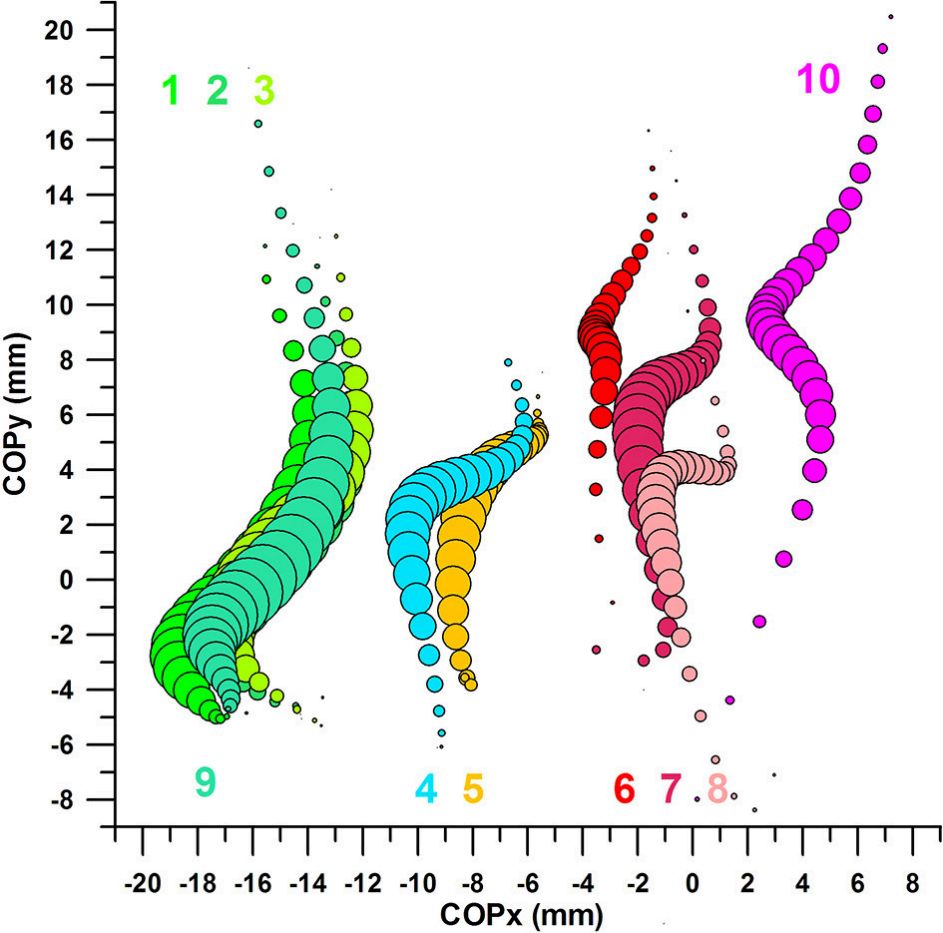
Centre of pressure in y-direction (COPy) against Centre of pressure in x-direction (COPx); the bubble size of the 10 bubble plots corresponds to the impact force; the graphs are aligned to the coordinate system of the sensor matrix: positive COPy data = distal; negative COPy data = proximal; negative COPx data = medial; positive COPx data = lateral; 1–8: cf. legend of Figure 11; 9: average COP of successful kicks, COPx, COPy, and Fmax within the favorable ranges; 10: average COP of successful kicks, COPx, COPy, and Fmax within the *un*favorable ranges.

Figure 12 shows two further datasets:

- dataset 9: average COP of successful kicks; COPx, COPy, and Fmax within the favorable ranges
- dataset 10: average COP of *un*successful kicks; COPx, COPy, and Fmax within the *un*favorable ranges.

The average COP paths of successful (dataset 9, Figure 12) and unsuccessful (dataset 10, Figure 12) kicks were clearly separated; the COPx data at Fmax (medio-laterally) by ~19 mm (Figure 11); and the COPy data at Fmax (proximo-distally) by ~9 mm.

#### Comparison of plots of COP paths

**Figure 13:**
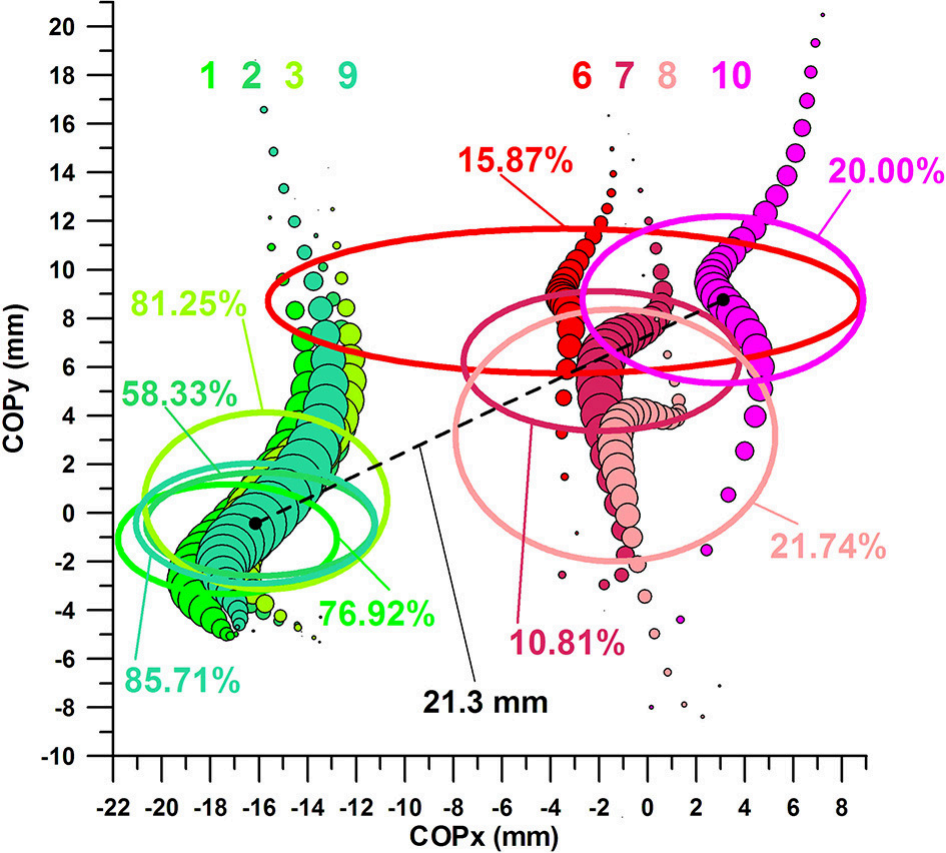
Sweet spot and dead spot; ellipses [one standard deviation about the average (black dots in the center of ellipses of sets 9 and 10)] refer to COP location at Fmax.

The four COP locations (green in Figure 13) of optimized parameters (favorable range) and successful kicks, i.e., datasets 1–3 and 9, are identical (*p* > 0.05) and perfectly superimposed.

The COPx values of the three 2-parameter combinations and one 3-parameter combination showed a *p*-value of 0.8411 (Kruskal–Wallis rank sum test), and were therefore not significantly different from each other.

The COPy values of the three 2-parameter combinations and one 3-parameter combination showed a *p*-value of 0.5896 (Kruskal–Wallis rank sum test), and were therefore not significantly different from each other.

Fmax values of the three 2-parameter combinations and one 3-parameter combination showed a *p*-value of 0.1933 (Kruskal–Wallis rank sum test), and were therefore not significantly different from each other.

The four COP locations (red in Figure 13) of *un*-optimized parameters (*un*favorable range) and *un*successful kicks, i.e., datasets 6–8 and 10, however, are, in 2 cases, not identical and clearly separated.

Fmax values of the three 2-parameter combinations and one 3-parameter combination showed a *p*-value of 1.7306e-11 (Kruskal–Wallis rank sum test). The reason for this result was that Fmax was optimized in dataset 7, and therefore exhibited a higher average (*post-hoc* tests *p* < 0.001 for dataset 7 compared to datasets 6, 8, 9).

The COPx values of the three 2-parameter combinations and one 3-parameter combination showed a *p*-value of 0.0022 (Kruskal–Wallis rank sum test). The *post-hoc p*-value of dataset 6 vs. dataset 10 was *p* < 0.005, which are the two datasets that are furthest apart in the x-direction in Figure 12.

The COPy values of the three 2-parameter combinations and one 3-parameter combination showed a *p*-value of 0.0001 (Kruskal–Wallis rank sum test). The *post-hoc p*-value of dataset 6 vs. dataset 8 was *p* = 0.001, which are the datasets that are furthest apart in the y-direction in Figure 12 (this result does not include dataset 10 which had a slightly higher standard deviation than set 6, and therefore was insignificantly different from set 8).

#### Definition and position of the sweet- and dead spots

We define the sweet spot as the impact zone between ball and boot/foot that maximizes the chance of scoring a goal (with a curve ball in this case).

Equally, we define the dead spot as the impact zone between ball and boot/foot that minimizes the chance of scoring a goal (with a curve ball in this case).

Figure 13 shows the COP of datasets 1, 2, 3, 9 and 6, 7, 8, 10 superimposed on 6 ellipses, the center of which is located at the average COPx at Fmax and average COPy at Fmax, and the semi-major and semi-minor axes correspond to one standard deviation of COPx and COPy data, respectively. The location of the 4 ellipses of datasets 1, 2, 3, 9 illustrates the finding detailed in the previous section, namely that there is no significant difference between the COPx data of the 4 sets nor COPy data of the 4 sets.

The 4 ellipses of sets 1, 2, 3, 9 define the location of a sweet zone, specifically as all 4 ellipses are superimposed rather than separated. This sweet zone can be reproduced with any of the three 2-parameter datasets (1–3). The ellipse of the three-parameter analysis constitutes the actual sweet spot, the location of which is almost identical to the ellipse of set 2 (COPx and COPy in their favorable ranges). The sweet spot is located more medially and proximally than the dead spot.

The three ellipses of sets 6–8 are more separated and define the dead zone, on the lateral and distal side of the sweet spot. The ellipse of the three-parameter analysis (dataset 10) constitutes the location of the actual dead spot, which is further lateral and distal of the ellipse of set 7 (COPx and COPy in their unfavorable ranges).

Note that the distance between the centers of the two ellipses is merely 21.3 mm (Figure 13), a distance that decides between high and low chances of scoring a goal with a curved kick. These chances are, according to the three-parameter analysis, 86% in the sweet spot and 20% in the dead spot, the percentages of which are significantly different. However, the 20% value was not significantly different from all three-parameter combinations other than the one with all three parameters in their favorable ranges (85.71%; Figure 10). Hypotheses 3 and 4 were confirmed in terms of the existence of clearly defined sweet and dead spots.

## Discussion

To the authors' best knowledge, the present study was the first one ever on sweet and dead spots on the foot or boot that maximize and minimize the chances of scoring a goal, respectively. This paper hypothesized that a spot exists on the shoe upper or dorsum of the foot that, when kicking a ball at this very spot, would maximize the chances of scoring a goal. The main result of this study was that a sweet spot was found on the medio-proximal aspect of the foot kicking a soccer ball. In contrast, the location of the dead spot was seen to be more latero-distal.

The term “sweet spot” was used in soccer shoes for the first time, to the best of our knowledge, when the Air Zoom Total 90 III was introduced with a side lacing system. This design was supposed to enlarge the sweet spot, defined as “the area of the boot that makes contact with the ball when shooting” (Wilson, 2006). However, as detailed in the Results section, there are regions within the contact area that provide higher or lower kicking accuracy. The term “sweet spot” should therefore be confined to the position of the COP that is associated with the highest chance of scoring. It is obvious that there is no single spot on a boot or foot that guarantees a 100% success rate. The “sweet spot,” enabling players to maximize their chances of scoring a goal, should depend on at least two parameters: COPx and COPy. A third parameter, e.g., the kick force, can be considered if it correlates with the chance of success statistically and if it has a mechanically explicable influence on kicking accuracy. In curved shots, the higher the kick force (normal force), the higher is the friction force. The latter improves the spin rate of the ball, resulting in the Magnus Effect and the aerodynamic sideward force. The greater the latter, the more pronounced is the curve of the ball.

Even if a “dead spot” was found in this research, with all three parameters in their unfavorable range, the low success chances when hitting this spot were not significantly different from the success chances if at least one of the three parameters is in its unfavorable range. This fact explains the sweet spot as a multiparameter-dependent location, which is, therefore, more specialized than the dead spot, but also more difficult to achieve when kicking a ball. The dead spot would be better defined by the entire area outside the sweet spot, or confined to the part of the area outside the sweet spot, where any possible ball contact actually occurs. This corresponds to that area that is actually used by the players for kicking. The fact that the dead zone was more widespread than the concentrated sweet spot supports hypothesis 4.

The problem that arises in this paper is whether the sweet spot is success- or player-controlled. In essence, the data could be skewed toward the better players, and therefore represent the kicking style of only the successful players. In the worst case, the sweet spot could be dominated by only one specific player. There are two counterarguments that stand on this assumption:

1. The sweet and dead spots discovered in this study are two extremes. They depend on the pre-separation of successful kicks with parameters (COP, force) in the favorable range (that maximizes success) and unsuccessful kicks with parameters in the unfavorable range (that minimizes success). All other combinations (e.g., unsuccessful kicks with [some] parameters in the unfavorable range) are located in between these extremes and therefore result in inseparable (i.e., statistically insignificant) COP locations of kicks with hits and misses (Results section “Comparison of parameter data of all hits against all misses”; and Figures 11, 12). This pre-separation pulls good and bad COP locations apart so that they become sweet and dead spots. This pre-separation does not separate participants in the first place such that the sweet and dead spots are independent of participants, but dependent on the success of a kick. Any pre-separation of participants would require taking only successful kicks of participants that score in e.g., more than 75% of kicks, and compare them to unsuccessful kicks of participants that score in e.g., < 25%.
2. For example a participant cohort consists of six participants (A–F), the sweet spot is defined by the average COP position of participants A–C (successful kicks, favorable range of parameters) and dead spot is defined by the average COP position of participants D–F (unsuccessful kicks, unfavorable range of parameters). The question that arises now is: why do participants A–C share the same COP? This could either be a coincidence or be based on what participants A–C have in common. This common parameter would then be a higher success rate. The reason for this would be that the ball-to-foot contact in the sweet spot guarantees a higher success rate in the first place. The same principle becomes evident from Hennig's (2011) study, describing the results of 20 participants kicking with two different shoes, shoe C with better kick accuracy and contact points more medially and proximally, shoe B with worse kick accuracy and contact points more laterally and distally. The two different ball-shoe contact points, determined with a pressure sensor, reflect different levels of kick accuracy.

The participants in our study contributing to the sweet spot (from most to least contribution) were: 5, 3, 10, 6+7, 1+4+8; and to dead spot were: 1, 2, 9, 4, 8, 7, 10, 6, 3. It is evident that the contribution to the sweet spot was made more by participants with better kick accuracy than by participants with less kick accuracy contributing to the dead spot. However, participants with a better kick accuracy, contributing to the sweet spot, share the same average COP, as shown in Figure 13 (black dots in the center of ellipses of sets 9 and 10).

Interestingly, our study revealed the same contact point distribution with respect to kicking accuracy as Hennig's studies (Hennig et al., 2009; Hennig, 2011), namely that the contact point providing better kick accuracy (our sweet spot) is located more medially and proximally with respect to the one providing less accuracy (our dead spot).

The frontal plane curvature in the dorso-medial part of the forefoot is more horizontal at the forefoot center (aligned to the transverse axis of the body), and more vertical on the medial side of the forefoot (aligned to the longitudinal axis of the body). Thus, the tangent to this curvature becomes more inclined from the center (top) of the forefoot to its medial edge. Consequently, kicking a ball with a contact point located more on the medial side generates a more vertical spin axis of the ball. The more vertical the spin axis, the stronger is the Magnus effect and the more pronounced is the curved flight path of the ball. This is consistent with the observed outcome of all missed kicks in our study, with the ball ending up on the right side of the goal.

The data obtained from this study are true only for the cohort examined and cannot necessarily be extrapolated to professional soccer players. It could very well be that in professional players, the gap between sweet and dead spots is more pronounced. Equally, the chances of scoring a goal when kicking the ball at the sweet spot are expected to be higher in professional players. It is nevertheless remarkable that a significant difference between sweet and dead spot could be found (thereby establishing sweet and dead spots as such) in an amateur soccer cohort, consisting of players of different kick accuracy.

A limitation of the present study is that we cannot be fully certain that the sensor did not move while kicking. Yet, we controlled the sensor location by visual inspection and palpation after every kick. Further evidence that the sensor remained immobile was that the four ellipses of the sweet spot (Figure 13) were superimposed with insignificantly different COP locations at Fmax.

This study revealed that the wearable device used in this study (smart soccer boot) is not only suitable to measure the training load of kicking, but also to assess the consistency of kicking in terms of how close the impact points are located relative to sweet and dead spots. In the future, we envisage that a smart soccer boot with fully integrated pressure matrix displays, on its digital twin representation method, the distribution of impact points, their impact force, and success (hits/misses) in real time, while calculating the position of sweet and dead spots. This will add another angle to measurement and management of training loads. Furthermore, an instantaneous biofeedback informing athletes of relevant parameters (i.e., distribution of impact points, impact force and probability of success) can be used to improve players' kicking performance beyond the abilities of an experienced coach.

## Practical applications and conclusions

The hypothesized existence of sweet and dead spots on a boot or foot when kicking a soccer ball was confirmed; however, the data comparison of all hits and all misses proved unsuccessful for establishing sweet and dead spots. As a consequence of this result, the data of COPx, COPy, and Fmax were investigated whether or not they can be separated in favorable and unfavorable ranges by means of a new method. Accordingly, the sweet and dead spots were found based on the hypothesized favorable/unfavorable parameter ranges (center of pressure in x/y-directions and/or peak impact force). These ranges maximized/minimized the chances of scoring a goal. Kicking the ball with the sweet spot maximized the probability of scoring a goal (58–86%), whereas having the impact points at the dead spot/zone minimized the probability (11–22%). The sweet spot was rather concentrated, independent of which parameter combination was used (two- or three-parameter combination), whereas the dead spot, located 21 mm from the sweet spot, was more widespread. The sweet spot was located more medial and proximal than the more scattered dead spots.

Based on the parameters analyzed and the discovery of the sweet and dead spots, we believe that in the future, the Smart Soccer Boot will be able to improve players' kicking performance by real-time biofeedback. Future studies should examine the application of the smart soccer boot in other types of kicks and investigate the existence of sweet/dead spots similar to the present study. Additionally, the sensor needs to be implemented in a boot and real-time biofeedback methods have to be developed.

From a practical point of view, we believe that this would allow players to directly analyze and alter their kicking technique based on the biofeedback signals (Düking et al., 2017) in order to hit the ball with the herein established sweet spot to increase the probability of a successful kick. Consecutively, players likely could train without the necessity of a coach being present to improve their kicking technique.

## Author contributions

All authors listed have made a substantial, direct and intellectual contribution to the work, and approved it for publication.

### Conflict of interest statement

The authors declare that the research was conducted in the absence of any commercial or financial relationships that could be construed as a potential conflict of interest.
